# Metabolic Profiling of a CB2 Agonist, AM9338, Using LC-MS and Microcoil-NMR: Identification of a Novel Dihydroxy Adamantyl Metabolite

**DOI:** 10.3389/fphar.2020.575691

**Published:** 2020-09-30

**Authors:** Chandrashekhar Honrao, Xiaoyu Ma, Shashank Kulkarni, Vinit Joshi, Michael Malamas, Alexander Zvonok, JodiAnne Wood, Roger A. Kautz, David Strand, Jason J. Guo, Alexandros Makriyannis

**Affiliations:** ^1^ Center for Drug Discovery and Department of Pharmaceutical Sciences, Northeastern University, Boston, MA, United States; ^2^ MAK Scientific LLC, Burlington, MA, United States; ^3^ Barnett Institute for Chemical and Biological Analysis, Northeastern University, Boston, MA, United States; ^4^ Department of Chemistry and Chemical Biology, Northeastern University, Boston, MA, United States; ^5^ Protasis Corporation, Seabrook, NH, United States

**Keywords:** metabolite identification, LC-MS, micro-coil NMR, adamantyl metabolism, di-hydroxyl adamantyl metabolite, CYP3A4 metabolism, CB2 agonist, cannabinoid metabolism

## Abstract

Adamantyl groups are key structural subunit commonly used in many marketed drugs targeting diseases ranging from viral infections to neurological disorders. The metabolic disposition of adamantyl compounds has been mostly studied using LC-MS based approaches. However, metabolite quantities isolated from biological preparations are often insufficient for unambiguous structural characterization by NMR. In this work, we utilized microcoil NMR in conjunction with LC-MS to characterize liver microsomal metabolites of an adamantyl based CB2 agonist AM9338, 1-(3-(1H-1,2,3-triazol-1-yl) propyl)-N-(adamantan-1-yl)-1H-indazole-3-carboxamide, a candidate compound for potential multiple sclerosis treatment. We have identified a total of 9 oxidative metabolites of AM9338 whereas mono- or di-hydroxylation of the adamantyl moiety is the primary metabolic pathway. While it is generally believed that the tertiary adamantyl carbons are the preferred sites of CYP450 oxidation, both the mono- and di-hydroxyl metabolites of AM9338 show that the primary oxidative sites are located on the secondary adamantyl carbons. To our knowledge this di-hydroxylated metabolite is a novel adamantyl metabolite that has not been reported before. Further, the stereochemistry of both mono- and di-hydroxyl adamantyl metabolites has been determined using NOE correlations. Furthermore, docking of AM9338 into the CYP3A4 metabolic enzyme corroborates with our experimental findings, and the modelling results also provide a possible mechanism for the unusual susceptibility of adamantyl secondary carbons to metabolic oxidations. The novel dihydroxylated AM9338 metabolite identified in this study, along with the previously known adamantyl metabolites, gives a more complete picture of the metabolic disposition for adamantyl compounds.

## Introduction

There are many drugs on the market containing an adamantyl ring as a key structural subunit used for treatment of serious diseases such as Parkinson's (amantadine) (Crosby et al., 2003), Alzheimer's (memantine) (Reisberg et al., 2003), type2 diabetes (saxaglipitin, vildagliptin) (Ahren, 2009), influenza (rimantidine) (Jefferson et al., 2004), skin disorders (adapalene) (Thiboutot et al., 2005), among others. (Wanka et al., 2013) These types of compounds target a variety of receptors and enzymes of therapeutic interest, including cannabinoid receptors (CB1, CB2) (Lu et al., 2005; Dixon et al., 2010), NMDA receptors (Gilling et al., 2009), ion channels (Stouffer et al., 2008), epoxide hydrolase (Burmistrov et al., 2018), 11β-hydroxy steroid dehydrogenase type I (Ye et al., 2017), cholinesterases (Kwong et al., 2017), and tyrosine kinases (Kaur et al., 2005). In many of these cases, especially for cannabinoid receptors, incorporation of the adamantane moiety improves the compound’s binding properties through favorable hydrophobic interactions with key residues within the binding pocket. In addition, adamantyl substitution increases the molecule’s lipophilicity, which facilitates its transport across the biological membranes to improve oral bioavailability and brain penetration (Milanese et al., 2011; Spilovska et al., 2016). A number of adamantyl derivatives have high affinity for the cannabinoid receptors, while several of these compounds are also sold as potent psychoactive agents for recreational purposes under the names of designer drugs, or herbal incense products etc (Banister et al., 2013; Gandhi et al., 2013; Gandhi et al., 2015). As these compounds are classified as controlled substances in many countries, including the US, detecting their presence in biological fluids is a major challenge for law enforcement agencies. In most cases, the parent compound cannot be detected in biological samples due to rapid elimination or extensive metabolism and the metabolites often lack authentic standards for proof of presence (Brents and Prather, 2014; Knittel et al., 2016). In addition, potential bioactivity and toxicity of these bio-transformed products is often difficult to assess in absence of synthetic standards.

The adamantyl moiety is known to be the major site for metabolic oxidation in numerous *in vitro* and *in vivo* metabolism and pharmacokinetic studies, while the adamantyl bridgehead tertiary carbons are generally believed to be the preferred oxidation site for CYP mediated metabolism (Rohde et al., 2007; Gandhi et al., 2015; Mardal et al., 2018; Shrestha et al., 2018). Conversely, as we have earlier reported (Honrao et al., 2015; Honrao et al., 2016), we now provide detailed evidence that secondary adamantyl carbons can also be the primary site for oxidative metabolism. Parallel to our work, similar findings were also recently reported for AKB48 using synthetically derived monohydroxylated adamantyl metabolite (Wallgren et al., 2017; Wallgren et al., 2018). These monohydroxylated adamantyl metabolites can also further undergo oxidation to dihydroxy metabolites *in vitro* and *in vivo* extensively. However, from biological system, these metabolites are often difficult to isolate in large enough quantities for NMR structural characterization (Vikingsson et al., 2015). Thus, to the best of our knowledge, there have not been any NMR studies reported elucidating the structures of the dihydroxylated metabolites to date. Moreover, the synthesis of these subsequent metabolites is often challenging and resource intensive due to lack of commercial starting materials and enantiomeric separations resulting from new chiral centers at the sites of oxidations. However, if it were possible to garner structural information of these metabolites, it could be used as biomarkers to detect adamantyl compounds used in drug abuse or to screen the metabolites in terms of bio-activity and toxicity or, through lead diversification approaches, could even lead to novel metabolically stable drug candidates encompassing adamantyl moiety.

To accommodate for limited sample quantities, there has been significant improvement in NMR mass sensitivity over the past two decades. The most notable developments include 1, 1.7 or 3 mm cryogenically cooled probes and microcoil flow NMR probes (Olson et al., 1998; Wolters et al., 2002; Lane et al., 2015; Anklin, 2016) the latter of which are not widely reported for traditional drug discovery metabolite identification applications. NMR microcoil probes are made of solenoid coils wrapped around the NMR flow cell with significantly lower active volume (1–5 μL) and have been shown to be ~10 to 12 folds more mass sensitive compared to conventional 5 mm probes at the same field strength, (Olson et al., 2004; Hopson and Peti, 2008; Lin et al., 2008) making it an attractive option to follow LC-MS metabolite characterization. In addition, microcoils are much more affordable compared to cryoprobes and can be easily swapped with conventional “5-mm” probes on a shared NMR instrument without disrupting the existing hardware (Kautz et al., 2005; Schroeder and Gronquist, 2006). Thus, to compensate for sample size limitations in drug metabolite studies, the microcoil probe offers a more viable and practical alternative, especially when used in conjunction with the most sample efficient zero dispersion segmented flow (SFA) sample delivery approach (Kautz et al., 2005). Kautz et al. previously reported detection limits as low as 200 ng for alkaloid extracts using a micro scale LC-MS-NMR platform with segmented flow sample loading, further demonstrating the sensitivity & usefulness of this technique (Kautz et al., 2005; Lin et al., 2008; Gathungu et al., 2012).

AM9338, 1-(3-(1H-1,2,3-triazol-1-yl)propyl)-N-(adamantan-1-yl)-1H-indazole-3-carboxamide, is a potent and selective CB2 agonist (hCB2 Ki = 3 nM, mCB2 Ki = 2.3 nM), which was discovered at the Center for Drug Discovery at Northeastern University as a candidate for potential multiple Sclerosis treatment. Multiple sclerosis is a serious autoimmune neurodegenerative disease affecting over 2 million people worldwide and characterized by inflammation mediated central and peripheral nerve demyelination, affecting the normal function of brain, spinal cord and optic nerves (Arevalo-Martin et al., 2008; Docagne et al., 2008). In animal models of multiple sclerosis, CB2 receptor activation by CB2 agonists has been shown to ameliorate the disease progression by reducing the demyelination of the nerves, hypersensitivity and associated neuropathic pain (Fu and Taylor, 2015). In this paper, we are reporting metabolic profile and characterization of oxidative metabolic sites of AM9338 in liver microsomal preparations combining both LC-MS and microcoil NMR. Our results also highlight the use of microcoil NMR systems for the structural characterization of trace level metabolites present in biological matrices to avoid expensive scale up or synthesis.

## Materials and Methods

### Solvents, Reagents, and Chemicals

LC-MS grade acetonitrile (ACN), formic acid (FA) and HPLC grade water were purchased from Thermo fisher scientific (NJ, USA). Fluorinert (FC-43) was purchased from Synquest Laboratories (Alachua, FL). Deuterated chloroform (CDCl3), deuterated dimethyl sulfoxide (DMSO-d6), Potassium dihydrogen phosphate (KH2PO4), dipotassium hydrogen phosphate (K2HPO4), magnesium chloride (MgCl2), and β-Nicotinamide adenine dinucleotide 2′-phosphate reduced tetrasodium salt hydrate (NADPH) were purchased from Millipore-Sigma (St Louis, MO). All of the aforementioned chemicals were the highest purity commercially available. Stocks of 20 mg/ml pooled rat, human and mouse liver microsomes were purchased from BioreclamationIVT (Baltimore, MD). AM9338, M1a (hydroxy-metabolite of AM9338), and M1b (isomer of M1a) were synthesize at the Center for Drug Discovery (Northeastern University, Boston, MA).

### Microsomal Incubation Conditions for Metabolite Identification

AM9338 (50 µM) was incubated at 37 °C with mouse, rat or human microsomes (0.5 mg/ml) in 50 mM potassium phosphate buffer, pH 7.4. Samples were taken immediately after the addition of NADPH (final concentration 1 mM) and again after 30 and 60 min of incubation; the reaction was terminated through protein precipitation with ice cold methanol followed by centrifugation at 4000 *g* for 10 min. The resulting supernatant was analyzed using LC-MS/MS.

### Biosynthesis of Phase I Metabolites

In an Erlenmeyer flask, AM9338 (100 µM final concentration) was mixed with pooled mouse liver microsomes (1.0 mg protein/ml, final concentration) and 50 mM potassium phosphate buffer, pH 7.4, containing 5 mM MgCl_2_ for a final volume of 15 ml and pre-incubated at 37 °C for 3 min in a 60-rpm shaking water bath. The reaction was started by the addition of NADPH (1.5 mM final concentration), allowed to incubate for 2 h at 37°C, and stopped by the addition of methanol. After centrifugation, the resulting supernatant was evaporated at 35 °C under nitrogen in an N-EVAP 111 evaporator (Organomation Associates, Inc., Berlin, MA) until concentrated approximately 10-fold. Ethyl acetate was added to the remaining solution and vortexed for 5 min and this procedure repeated three separate times; each time, the top ethyl acetate layer was separated, pooled and evaporated to dryness. The dried extract was reconstituted in acetonitrile: water (50:50 v/v) for column purification. This biosynthesis reaction was repeated to generate enough quantities of each metabolite for NMR characterization.

### HPLC Purification of Metabolites

Reconstituted extracts from the microsomal incubations were injected on a Supelco Discovery column C18, 250 × 4.6 mm column (Millipore-Sigma, St Louis, MO) on Thermo Accela HPLC system (Thermo Fisher Scientific, Austin, TX). The column temperature was maintained at 35°C, the flow rate was 0.8 ml/min, the injection volume was 20 µl and UV detector was set at 300 nm, maximal absorbance wavelength for AM9338. Chromatographic separation of the parent compound and metabolites was achieved using mobile phases consisting of 0.1% formic acid in water (A) and 0.1% formic acid in methanol (B) starting with an initial isocratic hold at 10% B for the first 4 min followed by a linear gradient over the next 31 minutes to 90% B, holding for a 7-min washout before returning to initial conditions; total run time was 53 min. Multiple metabolite fractions were manually collected, pooled, checked for minimum 98% purity by HPLC, evaporated under nitrogen stream and reconstituted for NMR analysis. Impure fractions were re-purified and evaporated under nitrogen prior to NMR analysis.

### Sample Loading and Microcoil NMR Analysis

Samples were injected into flow NMR microcoil probe utilizing direct injection or segmented flow techniques and in present research we are reporting applicability of using both sample loading technique for structural elucidation of metabolites. The purified metabolites M1 and M2 were analyzed by Zero dispersion segmented flow (on 500 MHz NMR) and flow injection sample loading (on 700 MHz NMR) methods respectively.

The Zero dispersion Segmented Flow Analysis (SFA) system was set up on a Varian Inova spectrometer (Palo Alto, CA) with a 500 MHz magnet according to previously described methods (Kautz et al., 2005; Kautz et al., 2013). The sample loader was assembled by inserting a needle of a 25 µl Hamilton syringe (400 µm o.d.) into one end of a 45 cm-length polytetrafluoroethylene (PTFE) tubing (200 μm i.d., 360 μm o.d; Cole-Palmer, IL), and connecting 5 cm of perfluorooctane sulfonate (PFOS) coated polyimide-clad silica capillary (200 μm i.d/360 μm o.d) to the other end with a zero dead volume union (Kautz et al., 2005; Kautz et al., 2013). Before injecting the sample, the probe was once flushed with deuterated acetone and then prefilled with fluorocarbon (FC-43). The purified and dried metabolite fractions were dissolved in 10 µl of deuterated chloroform containing 0.1% TMS. The sample plug was made in the following sequence: 1) prefill the silica capillary tip with FC-43, 2) 4 µl of the sample was drawn into the PTFE tube through the capillary tip without introducing any air bubbles (confirmed by microscope observation); 3) Finally, additional FC-43 was drawn into the syringe up to 25 µl volume mark. The injection device containing a sample plug was mounted on a manual syringe pump and connected to the probe inlet. Then the sample, as a plug sandwiched between FC-43, was manually loaded into the microcoil NMR flow cell using a syringe pump. Once the sample is correctly positioned in the NMR coil, the syringe pump was stopped and NMR data was collected. The NMR was set to acquire single scans every 2 s. The delivery flow rate, sample plug volume, and delivery time was previously optimized by using shim standards (1%TMS in CDCl3).

For flow injection analysis, CTC-PAL autosampler (model HTS, LEAP Technologies, Inc., NC) is connected to a microcoil probe (Protasis/MRM Corporation, Savoy, IL) and installed on a Bruker AVANCE II 700 MHz spectrometer. The NMR is equipped with a gradient inverse probe with a 10-μl flow cell. Deuterated solvent is delivered to the autosampler and the probe using a low-volume high pressure pump system (HTSL, Protasis Corporation, Marlboro, MA). The purified metabolite fractions were pooled together, dried under nitrogen and redissolved in 10 μl of DMSO-d6 (containing 0.1% TMS as reference standard) and then loaded onto the microcoil probe using a CTC-PAL autosampler controlled by One-Minute NMR automation (Protasis, Savoy, IL). DMSO-d6 was used as the push solvent to deliver the 8 µl sample plug to the center of the NMR detection coil (40 µl of DMSO-d6 is delivered at 30 μl/min flow rate). Once the sample is positioned at the right place in the coil, shims were adjusted and NMR spectra were recorded. Based on the preliminary ^1^H NMR data, additional 2D homonuclear (^1^H-^1^H) (i.e. COSY, TOCSY, and NOESY) or heteronuclear (^1^H-^13^C) (e.g. HSQC) NMR experiments were set up as necessary.

### LC-MS Method for Metabolic Stability

Chromatographic separation of AM9338 and its metabolites was achieved using a Phenomenex Kinetex F5 column (4.6 × 150 mm, 100 Å pore size, 2.6 μm particle size) on a Thermo Accela HPLC (Thermo, San Jose, CA, USA) coupled to an API-4000 Q-Trap (AB Sciex, Concord, Ontario, Canada). Samples were eluted with mobile phases consisting of 0.1 % formic acid in acetonitrile (A) and 0.1 % formic acid in HPLC grade water (B) starting at 10% A for 2.6 min, ramping to 90%A over the next 9.4 min, and holding at 90%A for 2.8 min before returning to initial conditions for a total run time of 18.5 min; the flow rate was 0.8 ml/min and the autosampler and column oven were set to 4°C and 35°C, respectively. The mass spectrometer was operated in electrospray ionization (ESI) positive ion mode with the following conditions: ion spray voltage = 5,500 V; heater temperature = 550 °C; curtain gas = 20 psi; Q2 collision gas = set as high; source gas 1 (GS1) = 50 psi; source gas 2 (GS2) = 55 psi. High purity nitrogen was used as a source and collision gas and the compounds were detected in the multiple reaction monitoring (MRM) mode.

### Docking Studies

The docking calculations were performed using Schrodinger 2017-2 with OPLS3 force field (Harder et al., 2016). The crystal structure of CYP3A4 (PDBID: 4D7D) (Kaur et al., 2016) was retrieved from the PDB bank and processed with Protein Preparation Wizard (Hanhoff et al., 2002). The compounds, AM9338 and its mono-hydroxylated metabolite, were prepared in Ligprep (Schrödinger, 2017). Induced fit docking (Veerkamp and Zimmerman, 2001) was conducted on each compound where the binding pocket was centered on the centroid of the co-crystallized ligand in 4D7D, and extra-precision (XP) mode was applied in the glide re-docking step.

## Results and Discussion

### Microsomal Stability and Pharmacokinetic Properties of AM9338

The susceptibility of AM9338 to CYP enzymes or hydrolytic enzymes was tested at 37°C in human, mouse and rat liver microsomes, in the presence or absence of NADPH. Stability studies show AM9338 is stable (tested up to 2 h) in absence of NADPH for all three species of microsomes including mouse, rat and human, suggesting stability to hydrolytic enzymes i.e. esterases, amidases etc. However, in the presence of NADPH, AM9338 showed extensive CYP dependent metabolism across all the three-species tested. The disappearance of AM9338 in microsomes followed a first-order elimination kinetics with low elimination half-life (t_1/2_ < 4 min) and high intrinsic microsomal clearance of microsomal protein across all three species as shown in **Table 1**.

**Table 1:** Microsomal half-life and intrinsic clearance.

Species	t_1/2_ (min) (Mean ± S.D.)	CL_int_ (µl/min/mg) (Mean ± S.D.)
Human	3.3 ± 0.6	420 ± 93
Mouse	0.47 ± 0.05	2948 ± 282
Rat	1.67 ± 0.33	829.9 ± 136

The non-compartmental pharmacokinetic parameters following intravenous (2 mg/kg, i.v.) and oral (8 mg/kg, p.o.) dosing are presented in **Table 2**. Following intravenous administration, the AM9338 mean plasma concentration declined rapidly, generating a short terminal half-life (t_1/2_ = 16 min) and high systemic plasma clearance (CL) (75.9 ml/min/kg, i.e., 85% of hepatic blood flow). The volume of distribution of AM9338 was 1.76 L/kg, which is greater than the total body water for mouse, suggesting that the compound is potentially sequestered in the tissues rather than remaining in the blood stream. Following oral dosing (8 mg/kg), oral bioavailability of ~15% was attained with the maximum AM9338 plasma concentration (Cmax: 0.24 µg/ml) was attained after 60 min post dosing. The poor metabolic stability, low oral bio-availability and high *in vivo* plasma clearance suggest susceptibility of AM9338 to hepatic or extrahepatic cytochrome P450-mediated metabolism *in vivo*.

**Table 2:** AM9338 pharmacokinetic (PK) parameters.

PK Parameters	I.V. (2 mg/kg)	Oral (8 mg/kg)
AUC (min × µg/ml)	26.3 ± 2.04	16.0 ± 8.2
CL (ml/min/kg)	75.9	489
Vd (L/kg)	1.76	79.9
t1/2 (min)	16.1	113
C0 (IV)/Cmax (Oral) (µg/ml)	2.11	0.239 ± 0.230
tmax (min)	–	60
Bioavailability (%)	–	15.5%

Mice (n = 3) were dosed with AM9338 either IV (2 mg/kg) or by oral gavage (8 mg/kg). Blood taken at various time points, immediately centrifuged for plasma, flash-frozen in liquid nitrogen and stored at −80°C prior to processing and analysis by LC-MS/MS. Pharmacokinetic parameters were calculated using non-compartmental analysis in WinNonlin (sparse data setting).

Furthermore, in an attempt to improve the metabolic stability various structural modification on AM9338 scaffold were attempted, most relevant for discussion are the analogues with varying linker chain lengths, secondary versus tertiary carboxamide attachment on adamantyl moiety, different alkyl substitutions at the adamantyl bridgehead carbons and replacement of adamantyl with less bulky cyclopropyl ring. When these analogues were assessed for metabolic stability in the microsomal clearance assay (data not shown) none showed any significant improvement in metabolic stability (average t_1/2_ < 5 min), except, for the analogue where adamantyl was replaced with a less hydrophobic cyclopropyl moiety, had shown dramatic improvement in the microsomal stability (average t_1/2_ > 60 min), however at the expense of CB2 receptor binding affinity (> 1 μM).

It has been previously shown, that adamantane substitution is favorable for CB2 receptor binding affinity (Nettekoven et al., 2013) and for selectivity on the quinolone scaffold, most likely due to the favorable hydrophobic interactions between the adamantyl moiety and the CB2 binding pocket (Pasquini et al., 2008; Lamoureux and Artavia, 2010; Mallipeddi et al., 2017). In recently published CB2 crystal structure with the antagonist AM10257, adamantyl moiety was shown to extends toward helices II and III, establishing hydrophobic interactions with multiple phenyl alanine and histidine residues further stabilizing ligand-CB2 receptor complex (Li et al., 2019). While on the other hand, the high lipophilicity of adamantanes also makes them good CYP enzyme substrates and is susceptible to extensive oxidative metabolism (Jasys et al., 2000; Lamoureux and Artavia, 2010). Thus, in order to optimize the metabolic stability for AM9338, it was necessary to identify the exact sites of metabolic modification on the adamantyl moiety, which can then be selectively blocked with metabolically stable groups.

### Metabolic Profile of AM9338 in Liver Microsomes

**Figure 1:**
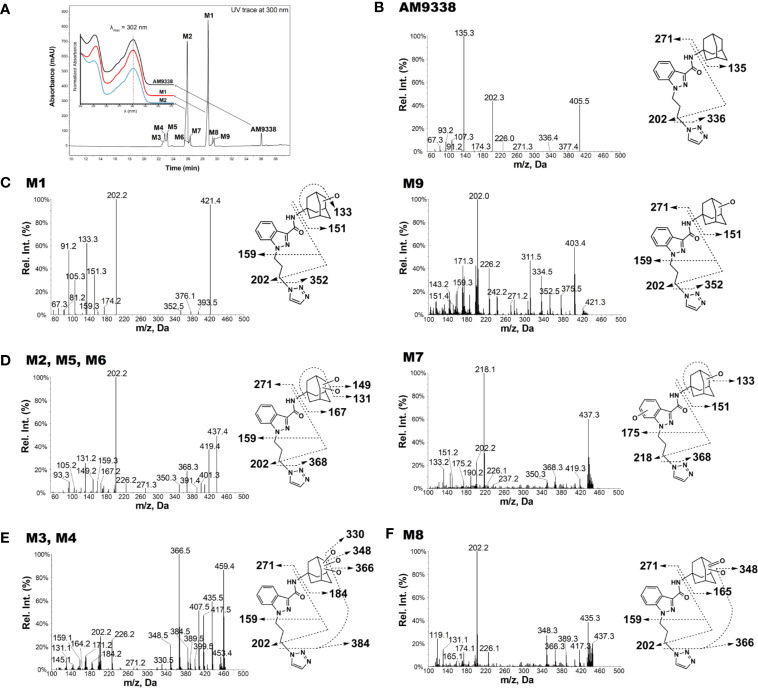
AM9338 and its metabolites MS2 spectra, fragmentation, and metabolite interpretation **(A)** LC-MS chromatogram depicting AM9338 metabolites generated 60 min after incubation with mouse liver microsomes. The inset contains the UV spectra for AM9338, M1 and M2 in black, red, and blue color traces, respectively. **(B)** MS2 spectra for AM9338. **(C)** MS2 spectra for monohydroxylated metabolites M1 and M9. **(D)** MS2 spectra for dihydroxylated metabolites M2, M5, M6, and M7. **(E)** MS2 spectra for trihydroxylated metabolites M3 and M4. **(F)** MS2 spectra for monohydroxylated-ketone metabolite M8.

AM9338 was almost completely metabolized after incubated at 37°C with pooled mouse liver microsomes in the presence of NADPH for 60 min. The LC-UV chromatogram from a sample at the end of the incubation period (**Figure 1A**) shows nine oxidative metabolites were formed; among them were two major and seven minor metabolites assigned as follows: Mono-hydroxylated (M1, m/z 421) and di-hydroxylated (M2, m/z 437) were major metabolites having the hydroxylations located on the adamantyl ring, and the minor metabolites consisting of two tri-hydroxylated metabolites M3 (m/z 453), M4 (m/z 453), as well as other oxidative metabolites M5 (m/z 437), M6 (m/z 437), M7 (m/z 437), M8 (m/z 435) and M9 (m/z 421). The metabolites M3 (trihydroxy), M4 (trihydroxy) and M8 (Ketone-hydroxyl) appear to be the metabolic end products of AM9338. These findings are consistent with previously published reports for other synthetic cannabinoids metabolites containing an adamantyl moiety (e.g. AKB-48, STS-135, and APINACA) (Banister et al., 2013; Gandhi et al., 2013; Gandhi et al., 2015). The estimated relative percentage of each metabolite was calculated using the UV peak areas at the end of the incubation: M1 (49%), M2 (37%), M3 (1%), M4 (5%), M5 (4%), M7 (3%), M8 (1%), and M9 (2%). In a time-course study, the formation of the oxidative metabolites was in the following order: M1> M2 >> M7, M9 ≥ M5, M6 > M3, M4, M8, suggesting that the adamantyl group is the most labile site on the scaffold. Human (HLM), Mouse (MLM) and rat liver microsomes (RLM) generated the very similar metabolite profile, with M1 and M2 being the major metabolic products, appearing in similar order of formation and relative quantities. Since mouse liver microsomes metabolized AM9338 faster than human liver microsomes, they were used to produce the M1 and M2 used in the LC-MS/MS and NMR studies.

### Characteristic AM9338 Fragmentation Pattern

The unique mass spectral fragmentation for AM9338 generates several product ion peaks (**Figure 1B**). Cleavage of the bond between the adamantyl carbon and the amide nitrogen generates the adamantyl cation (m/z 135) and the N-substituted indazole acylium ion (m/z 271). Alternatively, cleavage of bond between the alkyl chain terminal carbon and the triazole nitrogen leads to formation of a product ion at m/z 336, which then loses the adamantyl to generate the characteristic product ion at m/z 202. This characteristic product ion at m/z 202 generates additional daughter ions at m/z 174 and 159 in MS^3^ upon subsequent methylene loses from the alkyl chain. These diagnostic fragments were used to facilitate mass spectral interpretation of the unknown metabolites of AM9338.

### Mono-Hydroxylated Metabolites: M1 and M9

The most abundant metabolite (M1; m/z 421), which can be detected as early as 2 min into the incubation, is +16 da greater than AM9338 (m/z 405), indicating the oxidative addition of a hydroxyl moiety. The fragmentation pattern of M1 (**Figure 1C**) indicates that the position of hydroxylation is on the adamantane ring, as the masses of diagnostic fragment ions m/z 135 and 336 were increased by +16 da (m/z 151 and 352) in M1. This adamantyl hydroxyl group further undergoes elimination resulting in a H_2_O loss (−18 da) to generate the characteristic fragment ion at m/z 133. The presence of other M1 fragment ions (m/z 271, 226, 202, 174, 159) confirms absence of modifications on the N-substituted indazole moiety.

M9 (29.5 min), the other monohydroxylated metabolite of AM9338, retained longer than M1 (28.7 min) on the reverse phase column (**Figure 1A**), suggesting that it is slightly more lipophilic than M1; the ClogP of the two metabolites are 1.73 (M9) and 1.04 (M1) respectively. M9 produced similar fragment ions to M1, indicating hydroxylation on the adamantyl moiety (m/z 151 and 202). Unlike M1, the characteristic water loss fragment ion (m/z 133) was absent or of weak intensity in M9 spectra, indicating that the hydroxylation could possibly have occurred at the adamantyl bridgehead tertiary position.

### Di-Hydroxylated Metabolites: M2, M5, M6, and M7

M2, M5, M6 and M7 were identified as dihydroxylated metabolites with m/z 437, +32 da greater than the parent compound, AM9338 (m/z 405). After 60 min incubation time, among dihydroxylated metabolites, M2 (25.8 min) was the most abundant metabolite, generating fragment ions at m/z 131, 149, 167, 159, 271, and 226 (**Figure 1D**). This fragmentation pattern clearly suggests that both hydroxyl groups are present on the aliphatic adamantane ring. Subsequent fragmentation of the di-hydroxylated adamantyl cation (m/z 167) produced two characteristic fragments at m/z 131 and 149, indicating the loss of two –OH moieties (−18 water loss each).

Similarly, M5 (23.2 min) and M6 (26.2 min) produced the fragment ions m/z 368, 350, 131, 159, 271, 226, and 202, suggesting that both hydroxyls are located on the adamantyl moiety. When compared to M2 and M5, the metabolite M6 is retained on the reverse phase column longer, suggesting more lipophilic character and likely has both hydroxyls located at the adamantyl bridgehead (3° C-H) positions. This observation is further supported when the ClogP values, calculated by ChemDraw^®^ (PerkinElmer, Inc. Version 18.2), are compared: when one hydroxyl is at the secondary carbon (2° C-H) position and the second is at the 3° C-H position, the CLogP is −0.35; when both are at the 2° C-H, the CLogP is −0.38; but when both hydroxyls are at the 3° C-H, the resulted CLogP is 0.99 demonstrating that the higher CLogP values corresponds well with both hydroxyl groups are placed on 3° C-H position.

On the other hand, the M7 spectra (**Figure 1D**) contained fragments at m/z 368, 350, 151, 133 indicating that likely one hydroxyl is located on the adamantyl moiety and the second one is on the aromatic indazole moiety. Proof of the adamantyl hydroxylation is evident from the +16 modified fragments at m/z 218, 202, 190, 175. The key fragment at m/z 175, which is +16 da on the AM9338 alkyl chain fragment m/z 159, confirms the second hydroxyl placement on the indazole ring.

### Tri-Hydroxylated Metabolites: M3 and M4

Two trihydroxylated AM9338 metabolites (m/z 453, which is +48 da of parent) were identified: M3 had a retention time of 22.6 min and M4 had a retention time of 22.8 min. The MS^2^ spectra (**Figure 1E**) contained characteristic fragment ions at m/z 384, 226, 202, and 184, indicating that the three hydroxyl groups are all located on the aliphatic adamantyl moiety. Additional fragment ions at m/z 435, 417, and 399 correspond to the sequential loss of 1-, 2-, and 3-OH groups from the adamantyl moiety. These two metabolites are eluted earlier than other metabolites and could only be partially separated under the chromatographic conditions utilized.

### Monohydroxylation-Ketone Formation: M8

M8 mass spectra (**Figure 1F**) show a peak at m/z 435, which is consistent with monohydroxylation plus ketone formation. The presence of the characteristic fragment ions at m/z 226, 202, and 165 indicates that no modification occurred on the N-substituted indazole moiety, while the fragment ions at m/z 165, 366, and 348 confirms the presence of one hydroxyl and one ketone group on the adamantyl moiety. Fragment ions at 348 and 417 are the result of an –OH group loss (as -H_2_O) from the adamantyl moiety.

For 8 out of the 9 metabolites (except M7) we identified using LC-MS, the oxidation site(s) were located exclusively on adamantyl moiety. Interestingly, we did not find any metabolite bearing oxidative modification on the linker alkyl chain connecting the indazole and triazole moiety, nor in other AM9338 analogues with varying length of linker alkyl chain. On the other hand, AKB48, STS135 or APINACA, bearing no substitutions at the end of alkyl chain, undergoes oxidation at the free alkyl chain. These observations suggest the presence of aromatic group may protect the alkyl chain from CYP mediated oxidation.

Major metabolites of AM9338, M1 and M2 were selected for the NMR investigations to determine the exact metabolic site on the adamantyl. The photodiode array (PDA) UV spectra and LC/UV chromatograms for AM9338, M1 and M2 were presented in inset **Figure 1A**. The UV absorption characteristics of M1 and M2 were found to be identical to AM9338, thus allowed us to estimate quantities of the isolated M1 and M2 metabolites by comparing their UV peak under area values with that of parent compound standards, as previously shown by Selvan and Scatina et al. (Espina et al., 2009; Ravindran et al., 2011).

### Structural Characterization of M1 by Micro-Coil-NMR

**Figure 2:**
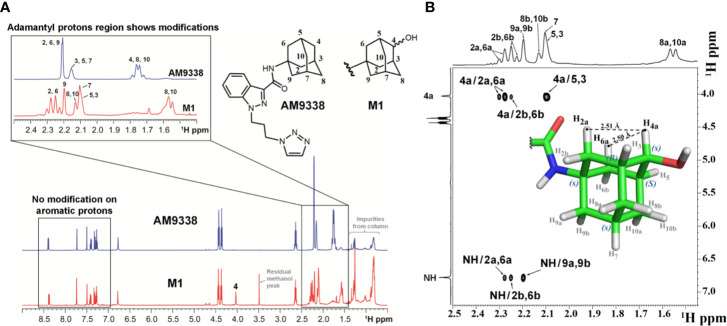
**(A)** Overlay of ^1^H-NMR spectra of AM9338 and its monohydroxylated metabolite (M1), **(B)** NOE NMR spectra and stereochemistry assignment for M1.

The metabolic oxidation site on M1 was assigned using ^1^H NMR spectra. 35 µg of M1 was isolated from microsomal preparations and purified using semi-preparative HPLC. M1 sample, as a 4 µl plug in CDCl3, transported in FC-43 into the micro-coil NMR probe using the SFA method as described in the method section. The resonances of the adamantyl protons on ^1^H-NMR spectrum in the 1.6- to 2.4-ppm region were assigned with the help of additional 2D experiments (^1^H-^1^H COSY, ^1^H-^1^H TOCSY, ^1^H-^1^H NOE, and ^1^H-^13^C HSQC). The detailed assignments are summarized in **Table 3**, along with the adamantyl proton numbering scheme depicted in **Figure 2B**. The chemical shifts of adamantyl protons are compared between AM9338 and M1: (1) The AM9338 proton signals of H2a, H2b, H6a, H6b, H9a, and H9b overlaps at 2.21 ppm as multiplet, While these protons in M1 shifted to give a broad signal at 2.20 ppm (br, 2H, H9a, and H9b) and the doublets in region 2.22 to 2.30 ppm (d, 4H, H2a, H2b, H6a, and H6b); (2) broad signal at 2.15 ppm (br, 3H, H3, H5, and H7; bridgehead protons) in AM9338, shifted upfield in M1 spectra at 2.09 ppm (m, 3H, H3, H5, and H7); and (3) the multiplet signal in AM9338 at 1.75 ppm (m, 6H, H4a, H4b, H8a, H8b, H10a, and H10b) was shifted to downfield region at 2.13 ppm as (m, 2H, H8b and H10b), a doublet at 1.56 (d, 2H, H8a, and H10a), and a triplet at 4.03 (t, 1H, H4a).

**Table 3:** ^1^H-NMR assignment of adamantyl protons for AM9338, M1, M1a, M1b, and M2.

Adamantyl protons	δ_H_ (*J* in Hz) in CDCl_3_			δ_H_ (*J* in Hz) in DMSO-d6	
	AM9338	M1/M1a	M1b	AM9338	M2
H2a	2.21 (m)	2.29 (d, 12.0)	2.43 (d, 12.0)	2.14 (br)	2.12 (m)
H6a					
H9a		2.20 (br)	2.24 (br)		
H2b		2.24 (d, 12.0)	2.06 (d, 12.0)		
H6b					2.21 (d, 11.7)
9b		2.20 (br)	2.24 (br)		
H3	2.15 (br)	2.09 (m)	2.16 (br)	2.11 (br)	2.12 (m)
H5					2.02 (br)
H7			2.08 (m)		
H4a	1.75 (m)	4.03 (t, 2.7)	–	1.71 (br)	3.87 (br)
H8a		1.56 (d, 11.7)	1.87 (d, 12.5)		
H10a					1.39 (d, 13.0)
H4b		–	3.84 (t, 2.7)		–
H8b		2.13 (m)	1.68 (d, 12.5)		
H10b					2.34 (d, 13.0)

The chemical shifts are expressed in δ ppm relative to tetramethylsilane (TMS) as the internal standard. Doublet, triplet, multiplet and broad signals are abbreviated as d, t, m and br respectively.

The ^1^H NMR spectra of M1 and AM9338 (**Figure 2A**) show the differences in the upfield aliphatic regions (1.5–2.4 ppm) which confirm the modification of the M1 adamantyl moiety. In M1, H4a peak moved downfield to 4.03 ppm, while H4b was absent. These changes indicate the attachment of electronegative OH group and identified adamantyl C4 as the oxidation site. The downfield region (7.0–8.5 ppm) of the M1 and AM9338 spectrum is the same, confirming that there were no metabolic modifications to either the indazole, amide or triazole protons. Moreover, the chemical shifts of six methylene protons from the alkyl linker chain also remained unchanged in M1.

### Determination of the Stereochemistry of M1 by NOE

The hydroxylation of AM9338 at the secondary position on the adamantyl generates a pseudosymmetric carbon on M1, leading to the possibility of two *RS*-diastereomers (Fujita, 2008). The stereochemistry of the M1, isolated from microsomal preparation, was assigned using NOESY spectrum (**Figure 2B**). The NMR signals for the H2a, H2b, H6a, H6b, H7a, H7b, H8a, H8b, H10a, and H10b protons were based on the presence of their respective NOE signal with the amide (–NH) proton. Strong NOE signal observed between the anomeric proton H4a (4.0 ppm) with the adamantyl protons H2a (2.29 ppm) and H6a (2.28 ppm), while no NOE was observed between H4a and protons H8b and H10b. The results strongly support that the anomeric proton (H4a) in M1 oriented parallelly to H2a and H6a protons, but not H8b and H10b protons and thus pseudo asymmetric carbon (C4) in M1 was assigned to be in *S* configuration.

**Figure 3:**
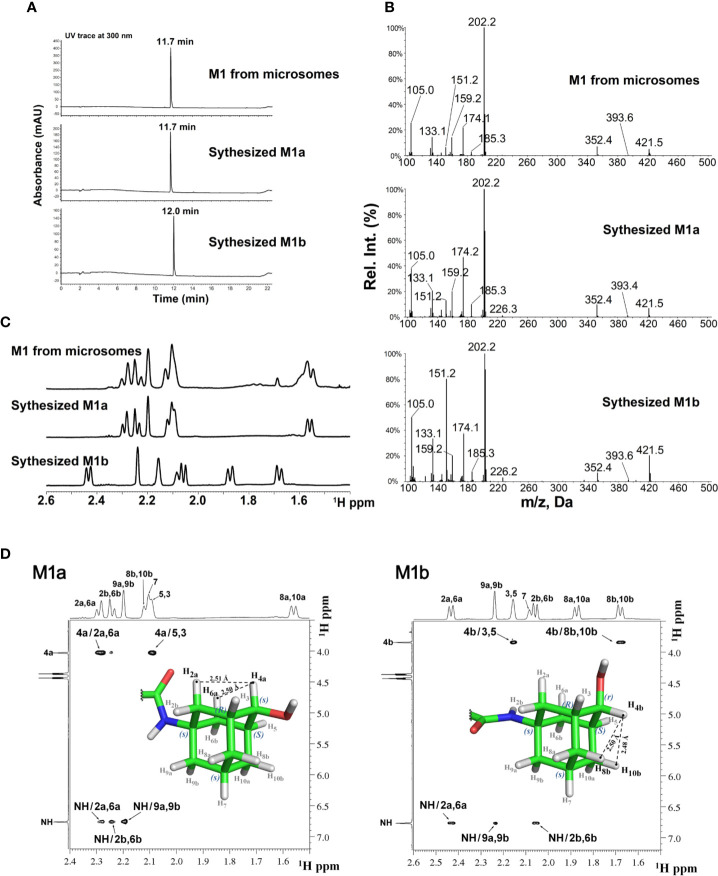
LC-MS and NMR characterization of M1 (microsomal metabolite) and its synthesized standards (M1a and M1b). **(A)** LC-UV trace for M1, M1a, and M1b; **(B)** MS2 spectra for M1, M1a, and M1b; **(C)** Overlay of ^1^H-NMR spectra of M1, M1a, and M1b; **(D)** NOE NMR spectra and stereochemistry assignment for M1a (left panel) and M1b (right panel).

The stereochemistry of the hydroxylated metabolite was further confirmed by chemical synthesis of M1 which generated two isomers, M1a and M1b from ketone reduction in the final step (synthetic scheme and isomer separation reported elsewhere). The LC retention times (**Figure 3A**) and NMR spectra (**Figure 3C**) of M1 isolated from liver microsomes were identical to the synthesized M1a isomer, but distinctively different from the M1b isomer. **Figure 3B** showed the comparison of MS2 spectra of M1, M1a and M1b, NOE spectra of M1a and M1b were also compared as shown in **Figure 3D**. Consistent with M1, strong NOE signals between the H4a (4.03 ppm) with H2a (2.29 ppm), and H6a (2.29 ppm) protons of synthesized metabolite (M1a) revealed their relatively close proximity (average separation distance of 2.53 Å) to each other. In contrast, NOE spectra for synthesized isomer (M1b) showed the strong NOE (short distance) between anomeric H4b proton (3.84 ppm) and adamantyl H8b (1.68 ppm) and H10b (1.68 ppm) protons, instead of H2a and H6a (**Figure 3D**). Based on these observations, the stereochemistry at adamantyl region was determined as (1*s*, 3*R*, 4*s*, *5S*, 7*s*) for the M1 or M1a and (1*s*, 3*R*, 4*r*, 5*S*, 7*s*) for the M1b, the synthesized isomer.

### Structural Characterization of M2

**Figure 4:**
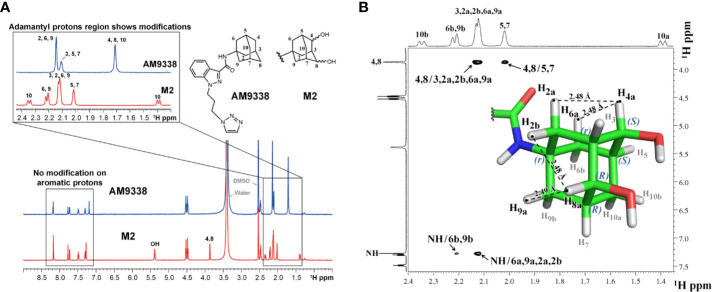
**(A)** ^1^H-NMR spectra overlay of AM9338 and its dihydroxylated metabolite (M2); **(B)** NOE NMR spectra and stereochemistry assignment for M2.

Approximately 40 µg of M2 was purified and isolated from mouse microsomal preparations using semi-preparative HPLC, evaporated, re-dissolved in 10 µl of DMSO-d6 and 8 µl of sample was injected into the micro-coil NMR as previously described in method section. The ^1^H NMR spectra comparison of AM9338 and the di-hydroxylated metabolite, M2 is depicted in **Figure 4A**. The differences observed in the aliphatic region (1.5–2.5 ppm) of the M2 spectra, confirms modifications on adamantyl moiety and the assignments of adamantyl NMR signals are summarized in **Table 3**. The differences between ^1^H NMR of AM9338 and M2 are as follows: (1) For AM9338, the signals of H2a, H2b, H6a, H6b, H9a, and H9b merged as a broad signal at 2.16 ppm. While in M2, four of them shifted to 2.12 ppm (m, 4H, H2a, H2b, H6a, and H9a) and remaining to 2.21 ppm (d, 2H, H6b and H9b); (2) The bridgehead proton signals of AM9338 overlapped as a broad signal at 2.11 ppm (br, 3H, H3, H5, and H7). In M2, H5 and H7 shifted to 2.02 ppm as a broad signal and H3 merged into the multiplet at 2.12 ppm; 3) H10a and H10b proton signals inside broad signal at 1.71 ppm in AM9338 spectra, were separated as two peaks at 1.39 ppm (d, 1H, H10a) and at 2.34 ppm (d, 1H, H10b) in the M2 NMR spectra; (4) The broad signal at 1.71 ppm in AM9338 spectra also contains the H4a and H8b signals, which shifted downfield to 3.87 ppm (br, 2H, H4a, H8a) in M2, and thus provides the strong evidence that C4 and C8 undergoes metabolic oxidation.

### Determination of the Stereochemistry of M2 by NOE

NOE experiments were performed to determine the stereochemistry at the two chiral carbons bearing hydroxyl groups in M2 (**Figure 4B**). The anomeric protons, H4a and H8a (3.87 ppm), have strong NOE cross peaks with H2a, H6a (average distance 2.53 Å), and H9a, H2b (average distance 2.53 Å,) respectively. The NOE spectrum reveals that H4a, H2a, and H6a are in the same side, while H8a, H2b, and H9a are in the same side. Based on these NOE signals carbon 4 and carbon 8 were assigned *S* and *R* configuration, respectively and the stereochemistry of M2 was determined to be 1*r*, 3*r*, 4*S*, 5*S*, 7*R*, 8*R*.

**Figure 5:**
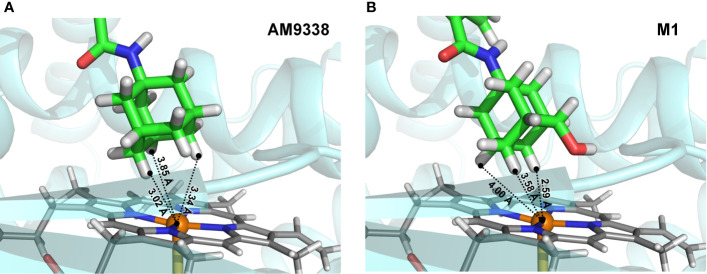
Docking poses of **(A)** AM9338 and **(B)** its monohydroxy metabolite M1, into CYP3A4 (PDB ID: 4D7D) binding pocket. Dashed lines represent distances between the heme iron center and the closest adamantyl hydrogens that may possibly be involved in the oxidation step. In both docking poses, hydrogens located on secondary carbons are much closer to the catalytic heme iron center than the three hydrogens on tertiary carbons of the adamantly moiety.

To further explain unusual susceptibility of secondary carbons of adamantyl moiety for metabolic oxidations, in spite of being chemically less reactive site, docking studies were conducted with AM9338 and metabolizing enzyme. Preliminary CYP phenotyping studies using CYP specific chemical inhibitors showed that CYP3A4 is the likely major CYP isoform responsible for the adamantyl oxidations in AM9338, in addition to the known role of CYP3A4 in oxidation of adamantyl moiety as reported earlier (Holm et al., 2015). Preliminary docking studies (**Figure 5**) of AM9338 and its mono-hydroxyl metabolite M1 with the CYP3A4 crystal structure (PDB ID: 4D7D) reveals that in both cases, the molecule orients itself in the binding site such that the hydrogens on the adamantyl secondary carbons 4, 8 and 10 come in closest proximity to the catalytic heme and thus are more accessible for oxidation over those on the tertiary carbons. These studies further support the argument that the site of oxidation on an adamantyl group is not only dependent on the most chemically reactive site but is also due to its stereochemical alignment of the ligand within the binding pocket of the oxidative enzyme. These findings possibly explain the unusual susceptibility of secondary adamantyl carbons on AM9338 to metabolic oxidations, which may likely also be the case for other adamantyl compounds with similar scaffold e.g. AKB48.

**Figure 6:**
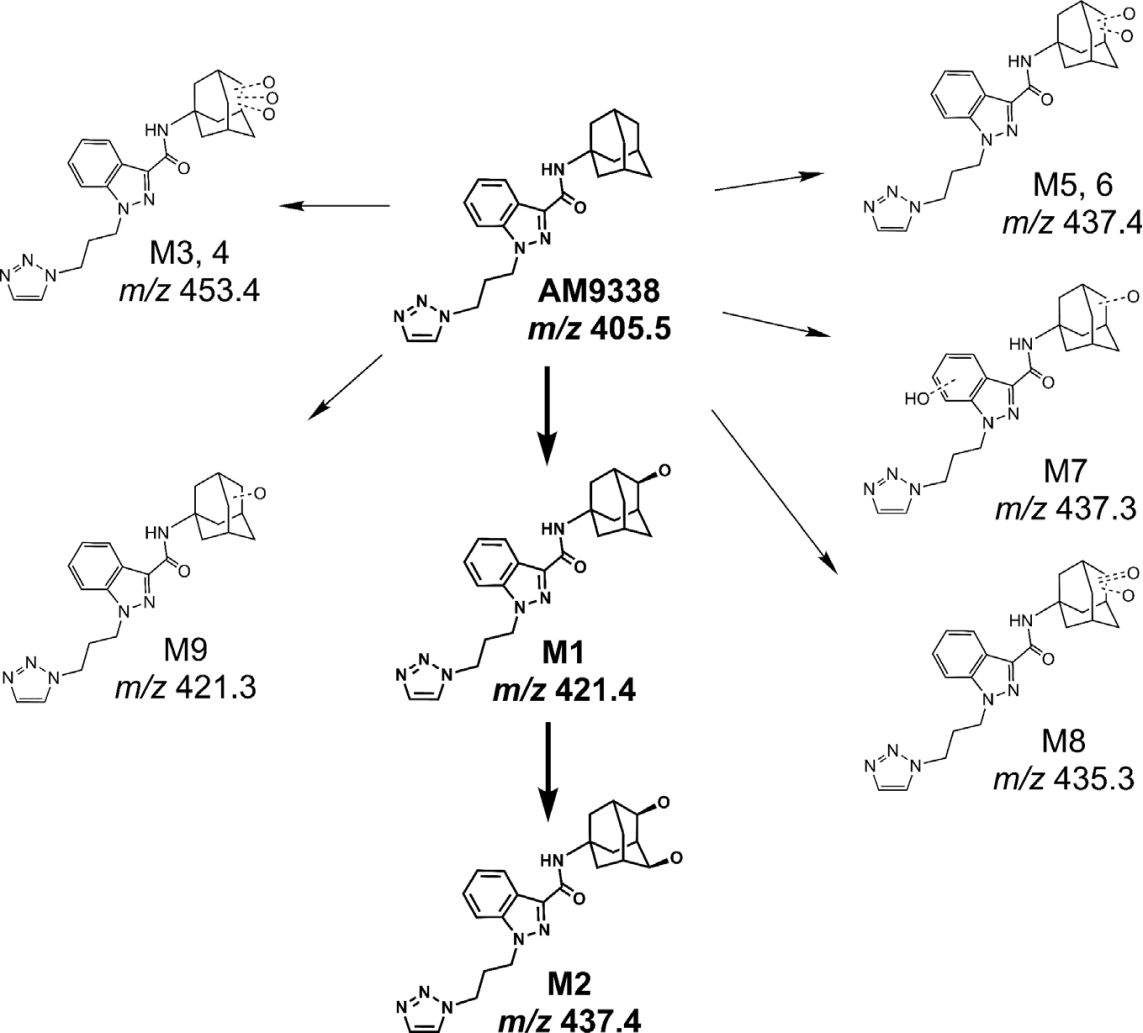
Proposed metabolic pathway of AM9338.

Metabolic investigations of AM9338 in human liver microsomes, revealed 2 major metabolites and at least 7 minor metabolites as identified by LC-MS, and the putative metabolic pathway is depicted in **Figure 6**. Out of these, the two major metabolites were further characterized using microcoil-NMR method, where the primary oxidative sites for both the mono- and di-hydroxyl metabolites are located on the secondary carbon(s) of the adamantyl moiety. Monohydroxy adamantyl metabolite with oxidation at secondary carbon site has been previously identified by our group as well as simultaneously reported by Wallgren et al. In their report, synthesis of monohydroxy metabolite was necessary for the structure elucidation of the metabolite using NMR, as quantities isolated from biological preparations (liver microsomes or urine) was insufficient for routine NMR characterization. Furthermore, adamantyls are known to undergo extensive metabolism giving rise to dihydroxylated metabolites and likewise we believed due to insufficient quantities and difficulty in their chemical synthesis, NMR based the structural studies were unfeasible and thus their structure remains unknown. We have purified this dihydroxylated metabolite from the liver microsomal preparations in microgram quantities and its structure was determined using microcoil-NMR analysis revealing both oxidation sites are located on secondary carbons of adamantyl moiety of AM9338. Further, using NOE correlation spectroscopy stereochemistry of this new metabolite has also been determined. Interestingly, metabolic stability studies of monohydroxy and dihydroxy metabolites of AM9338 revealed that dihydroxy metabolite is much more stable to the liver microsomes than monohydroxy metabolites (**Tables 4** and **5**), thus identifying both sites of metabolic oxidation on adamantyl could be a key towards blocking the metabolism of adamantyl moiety on AM9338 scaffold and likely on other similar scaffolds encompassing adamantyl moiety.

**Table 4:** Microsomal half-life and intrinsic clearance of M1 (monohydroxylation).

Species	t_1/2_ (min) (Mean ± S.D.)	CL_int_ (ml/min/kg) (Mean ± S.D.)
Human	13.1 ± 2.48	121 ± 20
Mouse	2.02 ± 0.11	2728 ± 223
Rat	4.41 ± 0.31	565 ± 4.4

**Table 5:** Microsomal half-life and intrinsic clearance of M2 (dihydroxylation).

Species	t_1/2_ (min) (Mean ± S.D.)	CL_int_ (ml/min/kg) (Mean ± S.D.)
Human	30.8 ± 6.7	54 ± 4
Mouse	10.5 ± 0.64	520 ± 54
Rat	26.8 ± 6.6	93 ± 10

While the present work focuses on the structure elucidation of these two major metabolites of AM9338, more NMR studies on the minor metabolites, *i.e.* M3 - M9, is currently underway to further confirm the likely metabolic sites as predicted from their mass spectral fragmentation pattern described in previous sections.

## Conclusions

We have applied LC-MS and micro-coil NMR to study the liver microsomal metabolites of our lead CB2 agonist, AM9338, a candidate compound with the potential for the treatment of multiple sclerosis. For the 9 oxidative AM9338 metabolites identified in this study, we found that mono- or di-hydroxylation of the adamantyl moiety was the primary metabolic pathway. Further microcoil-NMR characterization allowed us to determine the structures of two of the major AM9338 metabolites at microgram quantities, which revealed that the primary oxidative sites are located on the secondary adamantyl carbons, in contrast to the generally believed tertiary adamantyl carbons. The stereochemistry of these two major metabolites was also determined. To our knowledge, the di-hydroxylated AM9338 metabolite (M2) represents a novel metabolite for adamantyl containing compounds which has not been reported before. Moreover, this novel di-hydroxylated metabolite was found to be significantly more stable to liver metabolic enzymes than the mono-hydroxylated metabolite, which can be a promising scaffold to accelerate further optimization of AM9338 for metabolic stability. The new metabolic sites identified in this study, along with the previously known adamantyl metabolites, provide a more complete picture of the metabolic disposition for adamantyl-containing compounds. This new information could be valuable in developing metabolically stable drug candidates containing an adamantyl moiety as well as generating authentic metabolite standards for the assessment of their potential efficacy and safety.

## Data Availability Statement

The raw data supporting the conclusions of this article will be made available by the authors, without undue reservation, to any qualified researcher.

## Ethics Statement

The animal study was reviewed and approved by The Northeastern University-Institutional Animal Care and Use Committee (NU-IACUC).

## Author Contributions

CH: Designed and conducted experiments. XM and VJ: Metabolite scale up and NMR experiments. JW: Conducted animal PK experiments and data analysis. SK, AZ, and MM: Synthesized compound for studies and metabolite M1 for confirmation. RK and DS: assisted in setting up the microcoil probe and troubleshooting. CH, XM, and JG: Analyzed data and wrote the manuscript. JG and AM: Principal investigators oversaw the project, provided lab resources,and edited the manuscript.

## Funding

This research was supported by National Institute on Drug Abuse (NIDA) grants DA003801 (AM), DA009158 (AM) and DA032020 (JG).

## Conflict of Interest

Authors AZ and AM are employed by MAK scientific LLC, and DS is employed by Protasis Corporation.

The remaining authors declare that the research was conducted in the absence of any commercial or financial relationships that could be construed as a potential conflict of interest
