# Corrigendum: Metabolic Profiling of a CB2 Agonist, AM9338, Using LC-MS and Microcoil-NMR: Identification of a Novel Dihydroxy Adamantyl Metabolite

**DOI:** 10.3389/fphar.2021.630274

**Published:** 2021-04-22

**Authors:** Chandrashekhar Honrao, Xiaoyu Ma, Shashank Kulkarni, Vinit Joshi, Michael Malamas, Alexander Zvonok, JodiAnne Wood, Roger A. Kautz, David Strand, Jason J. Guo, Alexandros Makriyannis

**Affiliations:** ^1^ Center for Drug Discovery and Department of Pharmaceutical Sciences, Northeastern University, Boston, MA, United States; ^2^ MAK Scientific LLC, Burlington, MA, United States; ^3^ Barnett Institute for Chemical and Biological Analysis, Northeastern University, Boston, MA, United States; ^4^ Department of Chemistry and Chemical Biology, Northeastern University, Boston, MA, United States; ^5^ Protasis Corporation, Seabrook, NH, United States

**Keywords:** metabolite identification, LC-MS, micro-coil NMR, Adamantyl metabolism, di-hydroxyl adamantyl metabolite, CYP3A4 metabolism, CB2 agonist, cannabinoid metabolism

Roger A. Kautz was not included as an author in the published article. The corrected **Author Contributions** Statement appears below.

The authors apologize for this error and state that this does not change the scientific conclusions of the article in any way. The original article has been updated.

